# MicroRNA-29c Acting on FOS Plays a Significant Role in Nonalcoholic Steatohepatitis Through the Interleukin-17 Signaling Pathway

**DOI:** 10.3389/fphys.2021.597449

**Published:** 2021-04-01

**Authors:** Chao Cai, Da-Zhi Chen, Han-Xiao Tu, Wen-Kai Chen, Li-Chao Ge, Tian-Tian Fu, Ying Tao, Sha-Sha Ye, Ji Li, Zhuo Lin, Xiao-Dong Wang, Lan-Man Xu, Yong-Ping Chen

**Affiliations:** ^1^Department of Infectious Disease, The First Affiliated Hospital of Wenzhou Medical University, Zhejiang Provincial Key Laboratory for Accurate Diagnosis and Treatment of Chronic Liver Disease, Hepatology Institute of Wenzhou Medical University, Wenzhou, China; ^2^Department of Gastroenterology, The First Hospital of Peking University, Beijing, China; ^3^School of Wenzhou Medical University, Wenzhou, China; ^4^Department of Infectious Diseases and Liver Diseases, Ningbo Medical Centre Lihuili Hospital, Affiliated Lihuili Hospital of Ningbo University, Ningbo Institute of Innovation for Combined Medicine and Engineering, Ningbo, China

**Keywords:** FOS, NASH, micro array, Th17/Treg cells, bioinformatics

## Abstract

Nonalcoholic fatty liver disease is the most common hepatic disease in western countries and is even more ubiquitous in Asian countries. Our study determined that TH17/Treg cells were imbalanced in animal models. Based on our interest in the mechanism underlying TH17/Treg cell imbalance in nonalcoholic fatty liver mice, we conducted a joint bioinformatics analysis to further investigate this process. Common gene sequencing analysis was based on one trial from one sequencing platform, where gene expression analysis and enrichment analysis were the only analyses performed. We compared different sequencing results from different trials performed using different sequencing platforms, and we utilized the intersection of these analytical results to perform joint analysis. We used a bioinformatics analysis method to perform enrichment analysis and map interaction network analysis and predict potential microRNA sites. Animal experiments were also designed to validate the results of the data analysis based on quantitative polymerase chain reaction (qPCR) and western blotting. Our results revealed 8 coexisting differentially expressed genes (DEGs) and 7 hinge genes. The identified DEGs may influence nonalcoholic steatosis hepatitis through the interleukin-17 pathway. We found that microRNA-29c interacts with FOS and IGFBP1. Polymerase chain reaction analyses revealed both FOS and microRNA-29c expression in NASH mice, and western blot analyses indicated the same trend with regard to FOS protein levels. Based on these results, we suggest that microRNA-29c acts on FOS via the interleukin-17 signaling pathway to regulate TH17/Treg cells in NASH patients.

## Introduction

The most common hepatic disease in western countries is nonalcoholic fatty liver disease, and this disease has become even more ubiquitous in Asian countries such as China and Japan based on the drastic improvements in the standard of living in these countries in recent decades (Yang et al., 2016). Fatty liver disease is deemed to be a reversible condition wherein large vacuoles of triglyceride fat accumulate in liver cells through the process of steatosis. An unhealthy diet combined with a lack of treatment will inevitably lead to further hepatic damage that includes nonalcoholic steatohepatitis, liver fibrosis, liver cirrhosis, or hepatocellular carcinoma (Ong and Younossi, 2007). Currently, no effective treatment for nonalcoholic steatohepatitis is available (Satapathy and Sanyal, 2015).

In a previous study, we determined that the TH17/Treg cells balance is skewed in a nonalcoholic steatohepatitis (NASH) mouse model (He et al., 2017). Based on this, we attempted to analyze gene expression levels between NASH patients and healthy individuals using bioinformatics tools. We aimed to identify the enrichment results of differentially expressed genes (DEGs), the potential microRNA targets these genes, and to design animal experiments to validate the results of bioinformatics analysis using polymerase chain reaction (PCR) and western blotting. To obtain more in-depth and reliable results, we also attempted to mine the key genes involved in NASH and the microRNAs that target these genes, and we verified the bioinformatics results by performing PCR and western blotting.

Common gene sequencing analysis was based on one trial on one sequencing platform, where only gene expression analysis and enrichment analysis were performed (Gupta et al., 2017). We compared different sequencing results from different trials on different sequencing platforms, and we used the intersection of these results to perform joint analysis to achieve increased reliability.

Previous studies have indicated that the disorder of immune cells (Treg and Th17 cells) in fatty liver disease may be regulated by microRNA-29c by acting on the FOS gene, thereby regulating liver damage and fat deposition. As NASH is closely associated with immunity, this study provides a novel approach for the prevention and clinical treatment of NASH.

## Materials and Methods

### Flow Cytometry Analysis

Peripheral blood mononuclear cells were isolated from liver samples using lymphocyte separation medium (TBDscience, Tianjin, China) and staining with anti-mouse CD4 FITC (eBioscience, CA, United States) and CD25 PE (eBioscience), and they were then cultured in fixation/permeabilization reagent for 40 min and subsequently stained with anti-mouse Foxp3 PE-Cyanine5.5 (eBioscience). Prior to the analysis of TH17 cells, FITC-labeled CD4 lymphocytes were analyzed. After incubating the cells with a blend of PMA/ionomycin (eBioscience) and BFA/Monensin mixtures (eBioscience), intracellular staining was performed using anti-mouse/rat IL-17 PE (eBioscience). Treg cells were defined as CD4+CD25+Foxp3+ T cells, and TH17 cells were defined as CD4+IL-17+ T cells. For flow cytometry analysis, the cells were stained with fluorescent-conjugated antibodies and analyzed using a BD FACSCalibur platform (BD Bioscience, CA, United States) according to the manufacturer’s instructions. The ratios of TH17 or Treg cells to total CD4+ T cells were analyzed using FlowJo software.

### Selection of Gene Expression Data Series

Gene expression data series were collected from the Gene Expression Omnibus (GEO)^1^. We chose the trial based on the following criteria: (1) Organism was Homo sapiens; (2) Experiment type was expression profiling by array; (3) Trials included biopsy-diagnosed NASH patients and healthy controls. (4) The series data included available matrix files. The first selected expression series was GSE89632 from Allard JP that was performed on an Illumina HumanHT-12 WG-DASL V4.0 R2 expression beadchip platform. This trial included 48 patients with nonalcoholic fatty liver disease (25 simple steatosis and 23 nonalcoholic steatosis hepatitis patients) and 24 healthy living liver donors as HC. Nine samples (5 simple steatosis and 4 nonalcoholic steatosis hepatitis) were excluded during quality control. The final analysis included 63 participants (20 simple steatosis, 19 nonalcoholic steatosis hepatitis, and 24 healthy control patients). The second selected expression series was GSE24807 that was obtained from Liu W and performed on the GE Healthcare/Amersham Biosciences CodeLink Human Whole Genome Bioarray platform. This trial included 12 biopsy-diagnosed nonalcoholic steatosis hepatitis patients and total RNA from 5 different subjects that was purchased from ADMET. These subjects were not diagnosed with any liver disease.

### Computed Matrix Data Processing

Gene expression matrix data normalization and DEG selection were performed using R (version 3.4.0). *P-*value: *p-*value from Benjamini and Hochberg (False discovery rate). The cut-off values for DEGs were | logFC| > 2 and adj. *P* < 0.01.

### Venn Diagram Mapping

Coexisting DEGs were identified using the Venn diagram mapped in the free website tool Bioinformatics and Evolutionary Genomics^2^.

### Gene Ontology (GO) Analysis

GO analysis was performed using the free webtool WebGestalt^3^ (Wang et al., 2017). *P*-value: *p*-value from hypergeometric test, cut-off value was *P* < 0.05.

### Network Analysis

Network analysis and constituent enrichment analysis were both conducted using the website analysis system NetworkAnalyst^4^. Network visualization processing was performed using Cytospace (V3.5.1) supported by the National Resource for Network Biology (Xia et al., 2015).

### Pathway Enrichment Analysis

The free webtool KEGG Mapper^5^ was used for pathway enrichment analysis (Kanehisa et al., 2017).

### Animal Modeling and Sample Collection

A total of 12 C57BL/6 mice (male, 8 weeks of age) were randomly divided into two groups that included a normal group that was fed methionine and a choline-deficient control diet (TP0020MS, Trophic, China) for 11 weeks and a NASH group that was fed a methionine and choline-deficient diet (TP0020M, Trophic, China) for 11 weeks. On week 12 of the trial, we sacrificed the mice by cervical dislocation after collecting blood from the tail vein. Liver and ileum tissues were then collected. All mice were treated according to the protocols reviewed and approved by the Institution Ethics Committee of the Wenzhou Medical University (Approval Document No. Wydw2017-0023).

### Liver Biochemical Analysis

Serum was collected and stored at −80°C until further use. Serum alanine transaminase (ALT)/aspartate transaminase (AST) levels were detected using an automatic biochemistry analyzer (AU5800, Beckman Coulter, United States). The analysis was performed by the clinical laboratory of the First Affiliated Hospital of Wenzhou Medical University.

### Hematoxylin/Eosin and Red O Staining

The liver tissues were fixed in 4% paraformaldehyde and embedded in paraffin. The embedded tissues were prepared into 4 μm-thick slices and stained with hematoxylin and eosin (H&E) and Red O according to the standard protocols. Each slide was measured using a digital image analyzer (KS400, Carl Zeiss Vision, Germany) by a technician blinded to tissue grouping in three randomly selected fields. Histological assessment was conducted by two clinical pathologists who were blinded to the experiment.

### Real-Time Reverse Transcription Polymerase Chain Reaction (qRT-PCR) Assay

Total RNA was isolated from mouse liver tissue using RNAsio (Aidlab Biotechnologies Co, Beijing, China). The reverse transcription reaction was conducted using the PrimeScript^TM^ RT reagent Kit (Takara, Otsu, Shiga, Japan). Real-time PCR was performed using a QuantStudio^TM^ 5 Real-Time PCR System (ThermoFisher, Waltham, MA, United States) to assess the expression of FOS and microRNA-29c. Expression values were compared using the delta-delta-ct method, and FOS and miRNA-29c were normalized using the reference genes GADPH and U6, respectively. The experiments were performed in replicates. The primers used were included: GADPH: AAGAAGGTGGTGAAGCAGG (forward), GAAG GTGGAAGAGTGGGAGT (reverse), FOS: CACGTCTTC CTTTGTCTTCACCTAC (forward), GCCTTGCCTTCTCTGA CTGCTC (reverse); U6: CTCGCTTCGGCAGCACATATA CT (forward), ACGCTTCACGAATTTGCGTGTC (reverse), microRNA-29c: CGGGCTAGCACCATTTGAAA (forward), CAGCCACAAAAGAGCACAAT (reverse), TAGCACCATT TGAAATCGGTTA (stem loop).

### Western Blot Analysis

Liver tissues were lysed in radioimmunoprecipitation assay buffer (RIPA) (Boyotime, Beijing, China). A total of 20 μg of each protein sample was denatured for 5 min in sample buffer and then transferred onto a polyvinylidene fluoride membrane (Immobilon-p, Darmstadt, Germany) after separation in 10% polyacrylamide gels. Membranes were blocked with 5% BSA in TBST [10 mM Tris–HCl (pH 7.5), 150 mM NaCl, and 0.05% Tween-20] at 25°C for 1 h and then incubated with the specific antibodies at 4°C for 12 h. The membranes were then washed and incubated with horseradish peroxidase-conjugated secondary antibody (1:5,000, Biosharp, Hefei, China) for 1 h at 25°C. Protein levels were determined by chemiluminescence using an enhanced chemiluminescence kit (Thermo, Massachusetts, United States). Immunoreactive bands were digitally captured as images through the use of Bio-RAD ChemiDoc XRS (Bio-RAD, California, United States). The density values of these images were measured using the software Image Lab (Bio-RAD, California, United States).

### MicroRNA Site Prediction

The microRNA target prediction tool TargetScan^6^ was used for microRNA target prediction. Site conservation is defined by conserved branch length with each site type possessing a different threshold for conservation, where 8mer ≥ 0.8, 7mer-m8 ≥ 1.3, 7mer-1A ≥ 1.6, and 6mer sites are always classified as non-conserved. The context++ score for a specific site is the sum of the contributions of 14 features. The context++ score percentile rank is the percentage of sites for this miRNA with a less favorable context++ score. The weighted context++ score accounts for the abundance of each 3′-UTR tandem isoform in which the site exists, as estimated from the UTR profile (a compendium of 3P-seq datasets from the same species). PCT is the probability of conserved targeting (Friedman et al., 2009; Agarwal et al., 2015).

## Results

### Flow Cytometry Analysis

**FIGURE 1:**
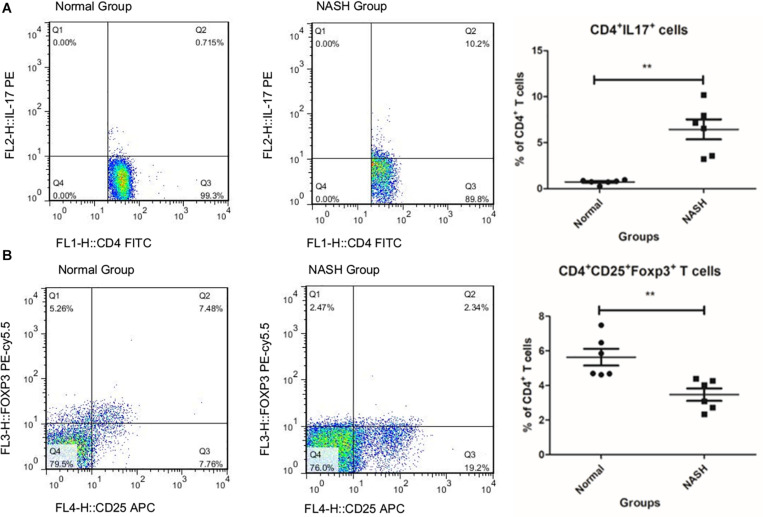
TH17 and Treg cell levels measured by flow cytometry. **(A)** Represents the ratio of TH17 to CD4+T in groups by flow cytometry analysis. **(B)** Displays the ratio of Treg cell to total CD4+T in groups **P < 0.01.

The ratio of Th17 cells to total CD4+ T cells in the NASH group was higher than that in the normal group (Figure 1A), while the ratio of Tregs in the NASH group was lower than that in the normal group (Figure 1B).

### Gene Expression Data Normalization and Identification of DEGs

**FIGURE 2:**
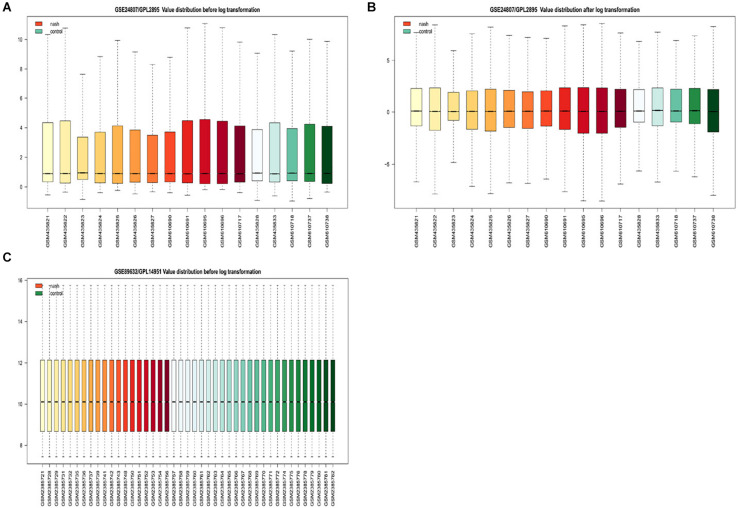
Examination of gene expression data normalization. **(A)** Shows GSE24807/GPL2895 Value distribution before log transformation, red for nonalcoholic steatosis hepatitis green for healthy control. **(B)** Shows GSE24807/GPL2895 Value distribution after log transformation, red for nonalcoholic steatosis hepatitis green for healthy control. **(C)** Shows GSE89632/GPL14951 Value distribution after log transformation, red for nonalcoholic steatosis hepatitis green for healthy control.

**FIGURE 3:**
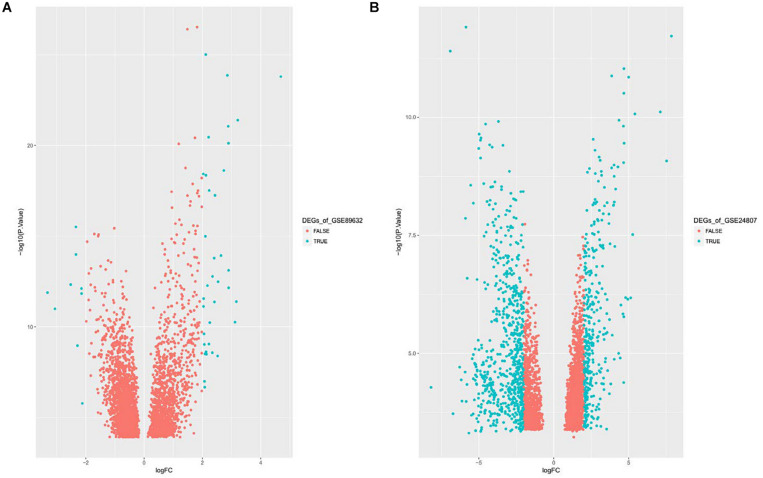
**(A)** Displays Volcano plot of DEGs of GSE89632, DEG: DEGs. **(B)** Displays Volcano plot of DEGs of GSE24807, DEG: DEGs.

Gene expression series matrices for GSE89632 and GSE24807 were analyzed using R script to examine the need for log transformation. Boxplots of the value distribution of samples both before and after log transformation are shown in Figure 2. As the matrix data for GSE89632 were normalized before output, it was not necessary to perform a log transformation, and a boxplot of GSE89632 after log transformation is therefore not presented. After log transformation, the value distribution of samples in GSE24807 was closer among the samples and exhibited an improved comparability for DEG mining. DEGs were identified using the cut-off values | logFC| > 2 and adj. *P* < 0.01. Volcano plots for DEGs from each gene expression series are presented in Figure 3. We identified 45 DEGs in GSE89632, and among these, 36 and 9 genes were up-regulated and down-regulated, respectively. According to the same method, 642 DEGs were identified in GSE24807, and among these, 152 and 435 genes were up-regulated and down-regulated, respectively.

### Identification of Coexisting DEGs in Both Gene Expression Series

**FIGURE 4:**
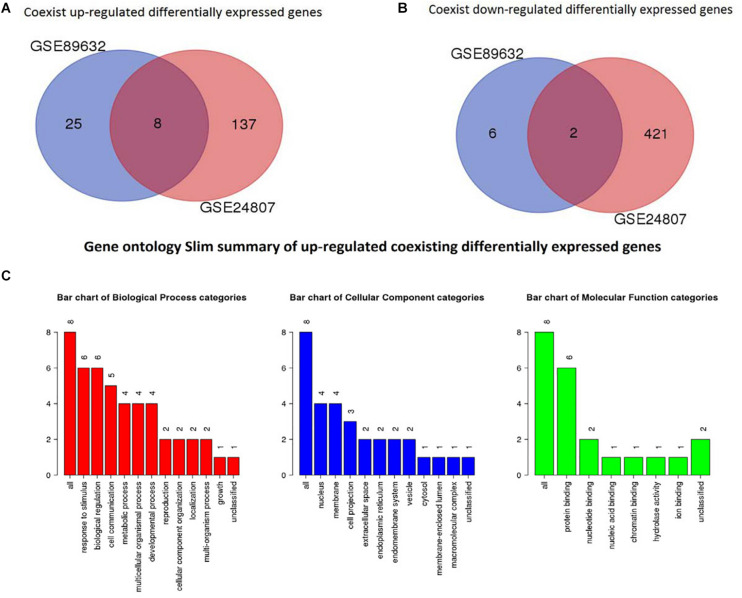
Identification of coexisting DEGs. **(A)** Shows Veen diagram of up-regulated DEGs. **(B)** Displays Veen diagram of down-regulated DEGs. **(C)** Shows gene ontology Slim summary of up-regulated coexisting DEGs.

To identify coexisting DEGs, we created Venn diagrams that are shown in Figure 4. We found that 8 differently expressed genes were up-regulated in both GSE89632 and GSE24807 and that 2 DEGs were down-regulated in both GSE89632 and GSE24807.

### GO Analysis of Coexisting DEGs

GO analysis was performed to identify potential supporting information regarding the importance of these coexisting DEGs. Using WebGestalt, we chose the enrichment results with a raw *p* < 0.05. As there were only 2 coexisting down-regulated DEGs, GO analysis was not performed. The GO Slim summary of up-regulated coexisting DEGs is presented in Figure 4. Further results from GO biological process enrichment using the WebGestalt Toolkit are described in Table 1. The biological process enrichment results suggest that the coexisting up-regulated DEGs are primarily involved in aging, feeding behavior, multicellular organism processes, and response to temperature stimulus. It was observed that FOS was involved in almost every process. Details from GO cellular component enrichment results obtained using the WebGestalt Toolkit are shown in Table 2. The 8 coexisting up-regulated DEGs were present in the microvillus membrane, cell projection, sarcoplasmic reticulum, terminal bouton, sarcoplasm, microvillus, axon terminus, neuron projection, cell projection part, and neuron projection terminus. The GO results of molecular functions provided by WebGestalt are shown in Table 3. The 8 coexisting DEGs were involved in functions that included insulin-like growth factor I binding, insulin-like growth factor II binding, RAGE receptor binding, and GTP binding.

**TABLE 1:** Gene ontology biological process enrichment results.

Biological process	Gene	*P*-value
Aging	*FOS; IGFBP1; CALCA*	0.000161
Feeding behavior	*FOS; CALCA*	0.000833
Female pregnancy	*FOS; CALCA*	0.002414
Conditioned taste aversion	*FOS*	0.002581
Multi-multicellular organism process	*FOS; CALCA*	0.003248
Response to temperature stimulus	*FOS; CALCA*	0.003436
Response to gravity	*FOS*	0.004298
Negative regulation of bone resorption	*CALCA*	0.005156
Neurological system process involved in regulation of systemic arterial blood pressure	*CALCA*	0.006013
Negative regulation of systemic arterial blood pressure	*CALCA*	0.006013

*P-value, p-value from hypergeometric test.*

**TABLE 2:** Gene ontology cellular component enrichment results.

Cellular component	Gene	*P*-value
Microvillus membrane	*S100P*	0.00687381
Cell projection	*FOS; S100P; CALCA*	0.017514333
Sarcoplasmic reticulum	*RASD1*	0.020503353
Terminal bouton	*CALCA*	0.022196031
Sarcoplasm	*RASD1*	0.022872419
Microvillus	*S100P*	0.026248515
Axon terminus	*CALCA*	0.037988405
Neuron projection	*FOS; CALCA*	0.038373505
Cell projection part	*S100P; CALCA*	0.039277758
Neuron projection terminus	*CALCA*	0.042651214

*P-value, p-value from hypergeometric test.*

**TABLE 3:** Gene ontology molecular function enrichment results.

Molecular function	Gene	*P-*value
Insulin-like growth factor II binding	*IGFBP1*	0.002934432
RAGE receptor binding	*S100P*	0.004032992
Insulin-like growth factor I binding	*IGFBP1*	0.004398954
GTP binding	*RASD1; ARL14*	0.006995867
Guanyl ribonucleotide binding	*RASD1; ARL14*	0.007800506
Guanyl nucleotide binding	*RASD1; ARL14*	0.007839841
R-SMAD binding	*FOS*	0.008052421
Insulin-like growth factor binding	*IGFBP1*	0.010239133
Receptor binding	*IGFBP1; S100P; CALCA*	0.010801787
RNA polymerase II core promoter sequence-specific DNA binding	*FOS*	0.020029692

*P-value, p-value from hypergeometric test.*

### Analytical Results of Protein-Protein Interaction Network Analysis and Gene Ontology Analysis of Genes in First-Order Networks

**FIGURE 5:**
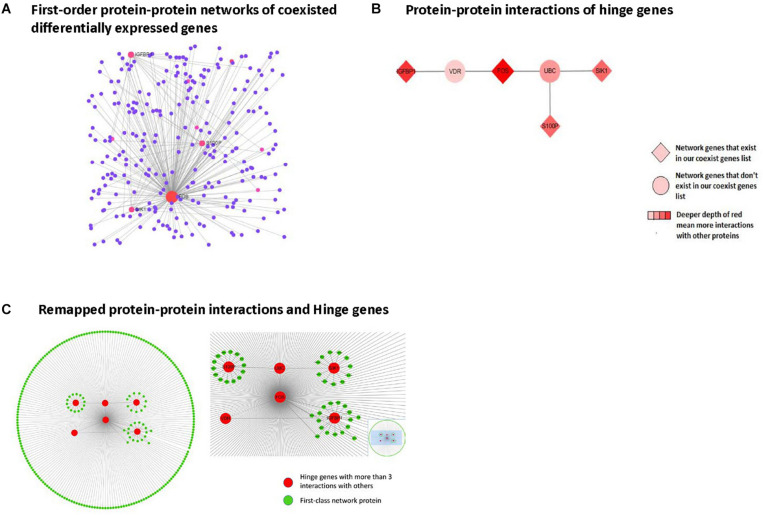
Protein-protein interaction network analysis in first-order networks and network hinge genes identifying. **(A)** Displays first order protein-protein interaction networks of coexisting DEGs, red spot means protein with more than three interactions with others. **(B)** Displays remapped protein-protein interactions and Hinge genes. **(C)** Displays protein-protein interaction of hinge genes.

Protein-protein interaction network analysis was performed using Network Analyst based upon results from the IMEx Interactome database. Zero-order networks were identified with fewer than 3 nodes (data not shown). First-order networks containing the coexisting DEGs and their interacting neighbors are shown in Figure 5. GO analysis results for the biological processes, cellular components, and molecular functions involving the first-order network genes are presented in Supplementary Tables S1–S3.

### Identification of Network Hinge Genes

From the first-class network results, we selected the hinge genes that interacted with more than 3 other genes and remapped the interaction layout using Cytospace. The remaining protein-protein interactions and hinge genes are shown in Figure 5. Among these, 4 genes (FOS, IGFBP1, SIK1, and S100P) were already present in our coexisting DEGs list.

### Pathway Enrichment Analysis of Hinge Genes

The KEGG mapper was used for pathway enrichment analysis of hinge genes. The results of the KEGG pathway analysis are shown in Table 4. As there were only 4 hinge genes used as input for analysis, all of the predicted pathways contained only 1 gene. With the exception of the glucagon signaling pathway involving SIK1, all the other pathways involved FOS.

**TABLE 4:** KEGG pathway analysis result.

KEGG pathway	Gene
Osteoclast differentiation	*FOS*
Rheumatoid arthritis	*FOS*
IL-17 signaling pathway	*FOS*
Amphetamine addiction	*FOS*
Choline metabolism in cancer	*FOS*
Th17 cell differentiation	*FOS*
Herpes simplex infection	*FOS*
Pertussis	*FOS*
Dopaminergic synapse	*FOS*
Salmonella infection	*FOS*
Estrogen signaling pathway	*FOS*
Leishmaniasis	*FOS*
Glucagon signaling pathway	*SIK1*
Fluid shear stress and atherosclerosis	*FOS*
MAPK signaling pathway	*FOS*
Prolactin signaling pathway	*FOS*
Th1 and Th2 cell differentiation	*FOS*
Pathways in cancer	*FOS*
HTLV-I infection	*FOS*
Hepatitis B	*FOS*
cAMP signaling pathway	*FOS*
T cell receptor signaling pathway	*FOS*
B cell receptor signaling pathway	*FOS*
Colorectal cancer	*FOS*
Toll-like receptor signaling pathway	*FOS*
Cholinergic synapse	*FOS*
Chagas disease (American trypanosomiasis)	*FOS*
Oxytocin signaling pathway	*FOS*
Breast cancer	*FOS*
Circadian entrainment	*FOS*
Apoptosis	*FOS*
TNF signaling pathway	*FOS*
Endocrine resistance	*FOS*

### Liver Biochemical Analysis

**FIGURE 6:**
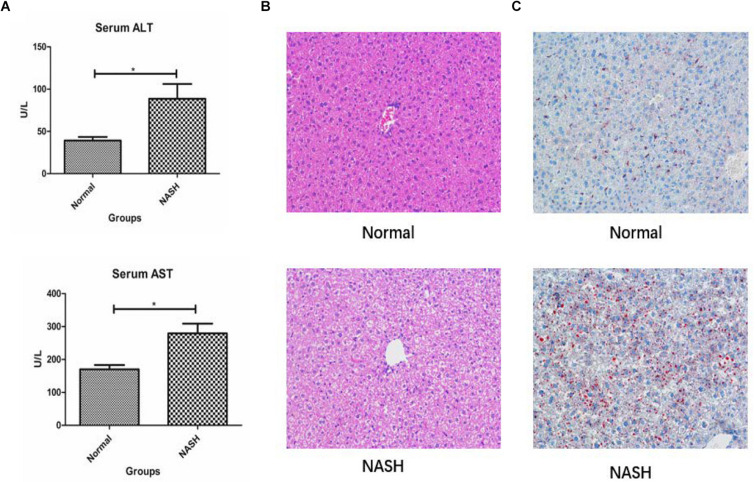
Liver biochemical analysis, HE and Red 0 staining. **(A)** Displays serum ALT and AST levels in Normal and NASH groups. **(B)** Displays HE staining of liver tissue. **(C)** Shows Red 0 staining of liver tissue *p < 0.05.

Liver biochemical analysis of the mice revealed that serum ALT and AST levels were significantly higher in the NASH group than in the controls (Figure 6A).

### H&E and Red O Staining

H&E staining revealed that the liver tissue structure of the NASH group was destroyed and that the liver cells exhibited balloon-like degeneration. Red O staining indicated that there was a certain degree of fat deposition within the livers of mice from the NASH group (Figures 6B,C).

### qRT-PCR and Western Blot Analyses of the FOS Gene

**FIGURE 7:**
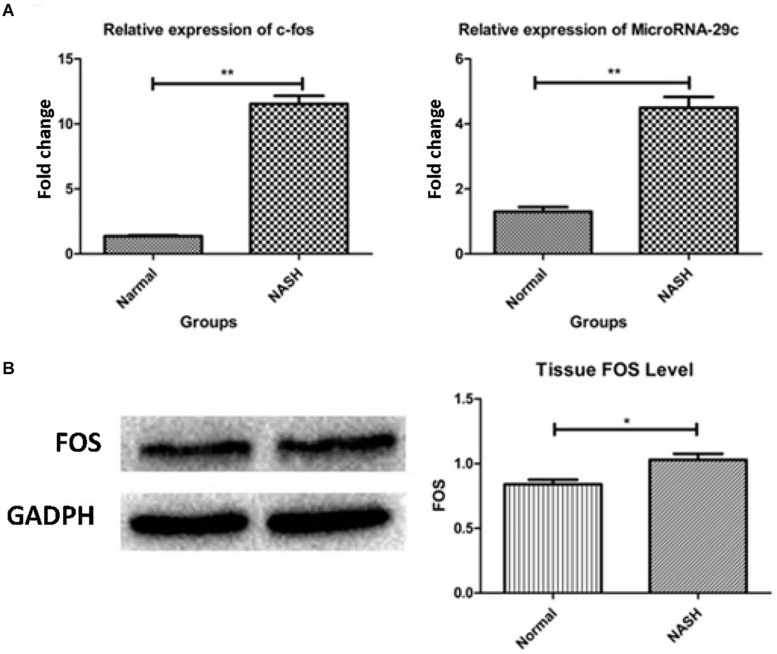
The expression of FOS gene and microRNA-29c. **(A)** Represents relative expression of FOS and microRNA-29c by RT-PCR assay. **(B)** Shows tissue FOS level by WB. *p < 0.05, **p < 0.01.

High expression of the FOS gene was observed in the NASH group according to qRT-PCR and western blotting analyses (Figures 7A,B).

### FOS MicroRNA Site Prediction and MicroRNA-Gene Interaction Network Analysis

**FIGURE 8:**
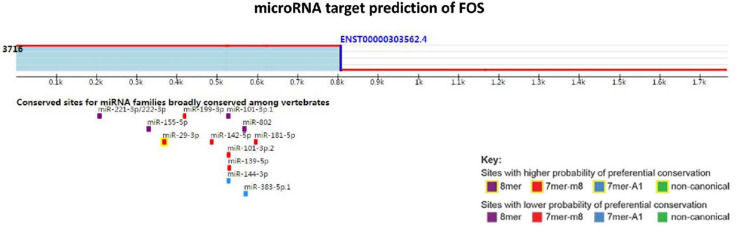
MicroRNA target prediction of FOS.

**FIGURE 9:**
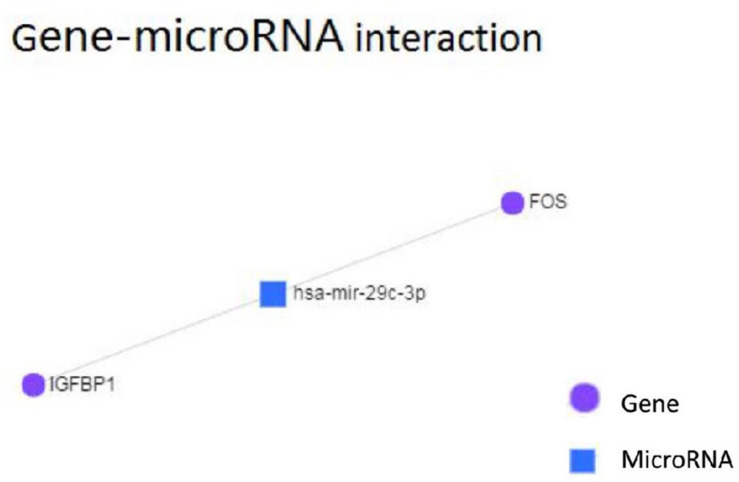
Gene-microRNA interaction analysis of hinge genes.

Using TargetScan for microRNA site prediction, 23 potential reverse microRNA sites and 566 adverse reverse potential microRNA sites were identified. The results of microRNA site matching are shown in Figure 8 and in Supplementary Table S4. MicroRNA gene interaction network analysis was performed using Network Analyst based on the TarBase and miRTarBase databases, and only one simple network of three nodes was identified. The miRNA hsa-mir-29c-3p only interacted with FOS and IGFBP1, as shown in Figure 9.

### qRT-PCR Analysis of MicroRNA-29c

High expression of microRNA-29c was observed in the NASH group according to qRT-PCR analysis (Figure 7A).

## Discussion

We observed that the ratio of Th17/Treg cells was unbalanced in the NASH mouse model (Gupta et al., 2017). To explore the correlation and mechanism of inflammatory cell imbalance in mice with nonalcoholic fatty liver disease, we identified important genes in the pathogenesis of nonalcoholic steatohepatitis through the use of gene expression sequencing and clustering analysis of gene sequencing. An animal model was established to verify the differentially expressed genes using PCR and western blotting. Additionally, microRNAs targeting these genes were further predicted by bioinformatics analysis and verified through PCR.

Traditional gene sequencing analysis is often used to assess a set of samples based on a sequencing platform, while single-sample analysis is one-sided. Therefore, we attempted to analyze the results of gene sequencing of different samples conducted on different sequencing platforms and to determine the coexistence of DEGs for bioinformatics analysis to allow for mutual verification and to achieve more reliable results (Xia et al., 2013). We screened the subjects from the GEO database for a human RNA sequencing trial w7hose living tissue was obtained from the liver. The experimental groups included a nonalcoholic steatohepatitis group and a healthy control group.

Using this filtering standard, we obtained two gene expression matrix data sets for GSE89632 and GSE24807 according to the screening. The first selected expression series was GSE89632 from Allard JP that was performed on an Illumina HumanHT-12 WG-DASL V4.0 R2 expression beadchip platform. The second selected expression series was GSE24807 that was obtained from Liu W and was performed on a GE Healthcare/Amersham Biosciences CodeLink Human Whole Genome Bioarray platform.

During the process of gene sequencing, sample differences, operation error, and deviation will all result in diminished signal strength among the samples. This may lead to a lack of uniformity in the value distribution of samples (Chen et al., 2003, 2016). Therefore, we used R software (version 3.4.0) to normalize two groups of experimental data through log2 transformation, and we implemented DEG screening after log2 conversion (if necessary). Based on our observation that the sequencing platform for GSE89632 processed normalization prior to matrix data output, there was no need for log2 transformation. In regard to GSE24807, after log2 transformation, the values of RNA expression in each sample became more concentrated, and this was more conducive for the screening of different genes.

We obtained a total of 45 DEGs from GSE89632, where 36 were up-regulated and 9 were down-regulated. We also screened 642 DEGs from GSE24807, where 152 were up-regulated and 435 were down-regulated. Upon observing the volcano plot, it was clear that there was a large difference in the number of differentiated genes that were identified from these two experimental results after sequencing on different platforms. We therefore extracted DEGs that co-existed in the two experiments by generating the Venn diagram. A total of 8 coexisting differentially expressed genes were up-regulated, and 2 DEGs were down-regulated. Using WebGestalt, we conducted a GO analysis on these 8 genes. As there were only 2 coexisting differentially down-regulated genes, we decided to explore each of them rather than ton use the bioinformatics analysis method. Here, we only analyzed the up-regulated coexisting DEGs. As shown in the chart, these 8 genes were involved in various biological processes that included aging, feeding behavior, female pregnancy, conditioned taste aversion, multicellular organism process, response to temperature stimulus, response to gravity, negative regulation of bone resorption, neurological system process involved in regulation of systemic arterial blood pressure, and negative regulation of systemic arterial blood pressure. Among these 10 biological processes, 7 processes involved the FOS gene, thus suggesting its importance. These 8 genes were involved in the organization of cellular components that included microvillus membrane, cell projection, sarcoplasmic reticulum, terminal bouton, sarcoplasm, microvillus, axon terminus, and neuron projection, and they were also involved in molecular functions that included insulin-like growth factor II binding, RAGE receptor binding, insulin-like growth factor I binding, GTP binding, guanyl ribonucleotide binding, guanyl nucleotide binding, R-SMAD binding, insulin-like growth factor binding, receptor binding, and RNA polymerase II core promoter sequence-specific DNA binding. Among these molecular functions, insulin-like growth factor binding function, GTP binding function, and RNA polymerase II core promoter sequence-specific DNA-binding functions reflect the associations between nonalcoholic steatosis hepatitis, cell growth, and RNA-DNA binding in some aspects.

To explore the possible interactions among these 8 genes, we used the Network Analysis online tool for protein-protein interaction analysis. By mapping the first-class interaction network, we obtained a potential interaction profile of the 8 genes and their direct interacting neighbors. In the first-class interaction network, we selected 7 hinge genes that exhibited more than three interactions with the other network members. Multiple interactions with other genes revealed that the 7 hinge genes involved in this interaction network consisted of DEGs in nonalcoholic steatosis hepatitis liver and their interaction neighbors. Among these 7 hinge genes, 4 were included in our identified DEGs. Based on these findings, we believe that IGFBP1, SIK1, and S100P, and especially FOS play significant roles in nonalcoholic steatosis hepatitis.

We next conducted pathway analysis using the KEGG Mapper. From these results, we observed that FOS was involved in almost every pathway. Among these pathways, the IL-17 signaling pathway was previously described as a damage factor in nonalcoholic steatosis hepatitis (Kisseleva, 2014). A previous study revealed that IL-17 is involved in the progression of nonalcoholic fatty liver disease to nonalcoholic steatosis hepatitis (Harley et al., 2014). Another also found that Th-17 cells are involved in alcoholic fatty liver disease and can be inhibited by LGGs treatment to facilitate milder steatosis.15 Researchers also previously determined that IL-17 may trigger hepatitis through Th-17 cells and cause hepatocellular carcinoma (Gomes et al., 2016).

Both H&E and Red O staining demonstrated that the mouse models used in our study successfully mimicked nonalcoholic steatohepatitis. Additionally, the experimental results obtained through modeling exhibit a certain correlation with the previous analysis. In animal experiments, real-time reverse transcription polymerase chain reaction analysis revealed that FOS was highly expressed in NASH mice, and western blot analysis demonstrated the same expression trend. This indicates that the action of the FOS gene is closely related to the development of nonalcoholic fatty liver disease.

MicroRNAs (miRNAs) are short (20–24 nt) non-coding RNAs that are involved in post-transcriptional regulation of gene expression in multicellular organisms due to their ability to affect both the stability and translation of mRNAs by targeting the 3′ untranslated region of mRNA (Lam et al., 2015). MicroRNAs have also been observed to be dysregulated in many diseases, thus revealing the importance of these molecules in the pathophysiology of several diseases (van Rooij and Kauppinen, 2014). Based on this, we aimed to ascertain the role of microRNAs in the context of nonalcoholic fatty liver disease. According to our KEGG pathway analysis results, FOS is involved in the IL-17 signaling pathway, and it also interacts with other DEGs and their other closely interacting neighbors. MicroRNAs that can regulate the expression of FOS may contribute to nonalcoholic steatosis hepatitis. The targets of microRNAs were predicted using TargetScan. Following the base complementary pairing principle, within FOS we found 23 potential conserved microRNA sites and 566 potential poorly conserved microRNA sites, among which has-mir-29 showed a higher probability of binding. It has been reported that down-regulated has-mir-29 may provide protection against NAFLD liver ischemia and reperfusion injury (Duan et al., 2017). A study also indicated that has-mir-29 can inhibit fibrosis in muscle tissues. These studies indicate that miR-29 possibly functions in suppressing liver fibrosis in NAFLD and NASH patients. We then tested our TargetScan results by performing microRNA-gene interaction network analyses on the hinge genes, and we observed that there was a network that consisted of only hsa-mir-29c-3p with FOS and IGFBP1 (Figure 8).

In animal experiments, qRT-PCR analysis confirmed the high expression of microRNA-29c in NASH mice. As microRNAs are described as gene suppressors, highly expressed microRNA-29c may be designed to inhibit the overexpression of the FOS gene. This suggests that microRNA-29c acting on the FOS gene is closely related to the development of nonalcoholic fatty liver disease.

Taken together, these results indicate that microRNA-29c acting on FOS may play an important role in NASH through the IL-17 signaling pathway in NASH patients, ultimately leading to an imbalance in TH17/Tregs inflammatory cell ratio. This explains the imbalance in inflammatory cells observed earlier in this study according to flow cytometry analysis. The pro-inflammatory cells associated with the FOS/IL-17 signaling pathway in the NASH group exhibited a significant increase in the percentage of Th17 cells in the blood. In contrast, the number of protective Tregs were reduced. The FOS/IL-17 signaling pathway may be a potential therapeutic target for liver fibrosis, a condition that is considered as an inflammation-mediating process in nonalcoholic steatosis hepatitis. Although it has been reported to be a biomarker for Alzheimer’s disease and type 2 diabetes, there are many studies that have explored the biomarker status of FOS in NAFLD, NASH, and even fatty liver disease. Moreover, the animal model experiments in our study have validated our bioinformatics analysis to some extent. This suggests that the FOS gene and IL-17 signaling pathway of NAS can be targeted to treat NASH by altering the expression of microRNA-29c; however, further research is required. Our research does possess several limitations. Due to the limited number of experimental human samples, we chose animal samples for verification. The ability of microRNA-29c to participate in NASH pathogenesis or of microRNA-29c intervention to improve steatohepatitis requires further study both in vivo and in vitro. Additionally, the ability of MicroRNA-29c and FOS to regulate TH17/Treg cells must be further confirmed. Regardless, our experimental results and analysis data provide new ideas for the treatment of NASH.

## Conclusion

In this study, we identified 36 up-regulated DEGs in GSE89632, 152 DEGs in GSE24807, and 8 coexisting up-regulated DEGs in both gene expression data series. Using protein-protein interaction network analysis, we identified 7 hinges, and 4 of these genes were included in our coexisting DEGs list (FOS, IGFBP1, SIK1, and S100P). Pathway analysis revealed that FOS was involved in the IL-17 signaling pathway, which impacts the nonalcoholic fatty liver disease process. MicroRNA target prediction and microRNA-gene interaction analysis revealed that microRNA-29c can potentially interact with FOS and IGFBP1 and possesses a conserved site with a higher probability of preferential binding to FOS. These findings suggest that FOS plays a significant role in nonalcoholic fatty liver disease, a process that is an important cause of inflammatory cell imbalance in NASH.

## Data Availability Statement

The datasets presented in this study can be found in online repositories. The names of the repository/repositories and accession number(s) can be found in the article/Supplementary Material.

## Ethics Statement

The animal study was reviewed and approved by the Ethics committee of Wenzhou Medical University.

## Author Contributions

CC, W-KC, L-CG, YT, S-SY, T-TF, and H-XT were responsible for substantial contributions to the study conception and design, data acquisition, data analysis, and interpretation. Y-PC, CC, X-DW, L-MX, Y-PC, and JL drafted the article and critically revised it for important intellectual content. All authors contributed to the article and approved the submitted version.

## Conflict of Interest

The authors declare that the research was conducted in the absence of any commercial or financial relationships that could be construed as a potential conflict of interest.

## Footnotes

1 http://www.ncbi.nlm.nih.gov/geo/

2 http://bioinformatics.psb.ugent.be

3 http://www.webgestalt.org/option.php

4 http://www.networkanalyst.ca/faces/home.xhtml

5 http://www.genome.jp/kegg/

6 http://www.targetscan.org/vert_71/
